# ‘To Cause Sleepe Safe and Shure’: Dangerous Substances, Sleep Medicine and Poison Theories in Early Modern England

**DOI:** 10.1093/shm/hkab064

**Published:** 2021-10-23

**Authors:** Elizabeth K Hunter

**Keywords:** sleep, laudanum, poison, recipes, Paracelsus

## Abstract

Recipes found in letters and manuscript receipt books testify to the use of potentially lethal substances in domestic sleep medicine. This article examines the theory behind the use of poisons to induce sleep, contrasting Galenic theory with the radical approach of the Paracelsians. According to Galenic medicine, the coldness of *stupefactives* such as henbane, deadly nightshade and the opium poppy were useful in counteracting fever and helping a patient to sleep. However, their coldness could also cause death. They were therefore used mainly in external medicine. The exceptions were *diacodium* made from native poppies that were considered less lethal, and sleeping draughts used in a surgical context. *Laudanum*, a new drug developed using alchemical methods to separate medicine from poison, broke with traditional safety advice. On account of its novelty, personal experience and recommendation were particularly important in establishing it within the canon of sleeping drugs considered safe for use.

Browsing the papers of Elizabeth I’s chief adviser and treasurer William Cecil, Lord Burghley, held in the British Library, the researcher comes across one that is striking in its domesticity. This is a letter from Lord Talbot, sent by a messenger carrying a gift of a box containing a substance Talbot’s wife has sent from her own store. Taken with a spoonful of claret wine, this drug promises to ‘procure sleep’. Bearing witness to its efficacy, Talbot writes that the ‘good effecte’ he has seen upon those who have taken it is such ‘as I never did see of any other thynge’. However, while he assures Burghley that he would not recommend it if he did not ‘know it to be a moste safe medesine’, he advises caution, suggesting that a trial should be made of the medicine by a third party before the great man takes it himself.^1^

Drug safety was a significant problem in early modern sleep medicine that has not so far received much attention in the secondary literature on the subject. The numerous recipes for sleeping aids that can be found in household collections from the time contain hints that finding a means of inducing sleep^2^ that was both safe and effective was a central concern. The instructions contained within these recipes can be linked to advice around drug safety found in medical guides and printed herbals. The letter to Burghley, however, contained a drug that was very different from the bulk of sleeping aids available at the time in that it did not follow the safety advice typically found in Hippocratic medicine. Dated 1588, it provides a rare insight into the early use of *laudanum—*a narcotic drug that first appeared in Europe in the sixteenth century. Before the tincture of opium and alcohol invented by Thomas Sydenham in the next century became popular, there were a variety of ways of preparing *laudanum*. That sent by Talbot was in solid form, measured in grains. Its appearance as a sleep remedy from this period onwards was the result of the impact of Paracelsian theories of poisoning, which challenged the Galenic hegemony that had governed the principles of sleep medicine for centuries. By examining early modern sleep recipes, and the context of early modern theories of poisoning that informed them, this article seeks to demonstrate just how radical this new drug was.

## Historiography and Sources

The importance of sleep in the history of health care is now well established in early modern scholarship. This began fifteen years ago with A. Roger Ekirch’s investigation into the numerous factors that could interfere with rest in this period, such as chronic pain, mental disturbance and environmental factors. More recently, Janine Rivière found 115 cases of patients suffering from sleep problems recorded in the notebooks of the physician Richard Napier and his associates.^3^ The work of Sasha Handley, Sandra Cavallo and Tessa Storey has demonstrated that, as one of the six non-naturals essential for the maintenance of health and prevention of disease, sleep was highly regarded in early modern households, with a significant amount of time, energy and resources invested in obtaining it.^4^ The work of Hannah Newton on convalescence has shown that sleep was important in recuperation from illness. Sound, uninterrupted sleep was believed to aid recovery, and acted as a sign of returning health.^5^

Part of the economy of sleep-management identified in this history are a wide variety of medicaments that were intended to aid the onset of sound sleep. The main evidence for this are manuscript household receipt collections, many of which contain recipes for sleep.^6^ Handley’s most recent paper touches on the issue of the potential danger such remedies posed, thrown into stark relief by an entry in a diary from 1599 recording the death of a physician by the self-administration of a sleeping medicine.^7^ Generally, however, studies of early modern slumber do not identify poisoning as a major limiting factor in attempts to medicate sleep. Nor do they situate the appearance of *laudanum* within the context of innovative approaches to handling poisons.

In addressing this question of safety in sleep medicine, I aim to deepen our understanding of early modern attitudes to dangerous medicines more generally. The question of how to turn poisons into medicines, and how medicines can cause poisoning, is a continuous theme in European history from ancient times to the present day, explored most recently in an edited volume by Ole Peter Grell, Andrew Cunningham and Jon Arrizabalga. The essays in this volume emphasise the importance of dose in determining whether a drug is therapeutic or harmful—an idea that is often attributed to Paracelsus, but can also be found in Galenic medicine.^8^ In sleep medicine, however, the most common method of reducing the possibility of poisoning was the use of external applications, in the form of compresses and aromatics, in order to avoid ingestion. It is in the context of warnings in printed literature against using dangerous ingredients in internal medicine, and the striking number of recipes recorded in manuscript receipt books that follow this advice, that we can fully appreciate the novelty of *laudanum*, and the controversy surrounding its development and use. The Paracelsian response to this controversy hinged on the theory that a process of treatment to remove noxious elements from a raw substance was an essential step to ensuring that a medicine was safe. As Frederick W. Gibbs argues in his 2018 monograph on poison treatises in the late medieval and early modern period, Paracelsus was actually extremely critical of physicians and drug makers who believed the danger of a medicine could be mitigated by simply scaling down the quantity.^9^

Studying sleep recipes from household collections in the context of these developing theories of poisoning can also provide further insight into the relationship between philosophies of nature, practical knowledge, and the recording and exchange of recipes, as discussed in the work of Pamela Smith, Elaine Leong, Alisha Rankin and others.^10^ The medical recipes in household collections cannot be read as an uncomplicated reflection of domestic practice. They were part of the tradition of collecting secrets, which had its roots in ancient and medieval esoteric writings.^11^ Determining what kind of knowledge secrets represent can be challenging. At one end of the spectrum are instructions that codify practical knowledge and experience, at the other, are highly unlikely formulas including impossible techniques or mythical ingredients. Recipes were often valued as a form of social exchange, and for the sense they gave the reader and collector that nature was accessible and malleable to the non-expert.^12^

Leong and Rankin have found that medical recipes were compiled primarily for the purpose of trialling, and that trials could be ambitious and experimental in nature.^13^ As Elizabeth Spiller has written of receipt collections, ‘the domesticity they represent is neither trivial nor safe’.^14^ Recipes do not confine themselves to minor ailments, but offer treatments for deep, badly infected wounds and scalds, ulcers, gall stones and cancerous swellings. They promise powerful cures that can save the patient from the violence of surgery, and miraculous recovery where the physician has failed.^15^ Some noble and gentlewomen acquired great skill and knowledge in pharmaceutical methods, and were famous for their panaceas and poison antidotes.^16^

The sleep recipes analysed in this article are taken from collections owned by the gentry and nobility of England, dating from the early seventeenth to the early eighteenth century. I discovered over 150 recipes relating to sleep in 52 manuscripts held at the Wellcome Library, the British Library and the Folger Shakespeare Library.^17^ They range from mild soporifics to strong sleeping draughts that would knock a patient out for hours. There are four recipes to make *laudanum* using alchemical methods and equipment. Some reflect the particular interests of their compilers. A collection by Jane Jackson entitled ‘A very shorte and compendious Methode of Physicke and Chirurgery’ contains a number of methods for reviving a ‘sleeping’ (unconscious) patient, and a powerful sleeping ‘drinke’ that was clearly intended to be used in a surgical context. The Boyle family collection, probably compiled by Robert Boyle’s sister, contains two different methods for making *laudanum*, reflecting the family’s interest in alchemical experiments.^18^ A very small number of sleep recipes are based on principles of natural magic.^19^ The bulk of the recipes are cures for sleeplessness, often in the context of sickness, pain or fever, based on Galenic principles.

## Probatum Est

A preoccupation with safety can be inferred from the titles and ‘tag phrases’ used in sleep recipes. One from Lady Ayscough’s collection involves placing warm bags of rue and grated nutmeg on the temples. This would not seem particularly dangerous, and yet the recipe ends with the assurance that ‘This is a safe meanes’.^20^ A recipe in Elizabeth Okeover’s collection is entitled ‘To cause sleepe safe and shure’.^21^ One kept by the family of Philip Stanhope, the Earl of Chesterfield, includes a recipe for a posset that was ‘safe for a woman with childe or lyinge in’.^22^ An entry in a collection by the naturalist Sir Hugh Plat, which involved taking four grains of *laudanum*, promised ‘To cause temperat sleep w^th^out hurt’.^23^

In the case of opiates, there appears to have been a concern about the dangers of overdose. Whereas most recipes are inexact about quantities and dosage, only sometimes giving measurements in spoons, opiates are quantified using minute units of measurement. Recipes involving *laudanum* specify the dose to be taken in grains or drops (depending on whether it was in solid or liquid form),^24^ in one instance differentiating between children and adults.^25^ In a collection begun by Elizabeth Jacobs in the mid seventeenth century, a recipe to procure sleep involves mixing half a grain of *laudanum* with mithridate into a pill: ‘Take noe more without good Advice’, the author of the receipt adds.^26^ One collection also contains an antidote in case of accidental overdose: ‘Cure for on that as taken Laudnum: Lemon Juice taken Emeaditly with is an Emitic and prevents the Lethargie appearance of Death and consequently preserves Life’.^27^

It is difficult to know on what basis these assurances of safety were made. Some receipt books contain notes or systems of markings in the margin to indicate which recipes had been tried, and there are also examples of recipes that have been crossed out because the results were dissatisfying.^28^ None of the sleep recipes I have found, however, have such annotations or marks; but they do contain what have been termed ‘efficacy phrases’, such as *probatum est* (‘it has been tried/tested’), ‘proved’, ‘approved’ or ‘approved a yoused’.^29^ There has been some debate over the significance of ‘efficacy phrases’, which appear in English recipes from the medieval period onwards.^30^ Some sleep recipes include the name or initials of the person who recommended it,^31^ but the phrase alone was not necessarily indicative of first-hand experience. What they do suggest is the empirical orientation of recipes, which were valued for their link with direct experience. As Alisha Rankin puts it, the indication that ‘*someone* had tried them’ [original emphasis] was reassuring for those involved in the practice of making and using medicines.^32^ The secondary literature on ‘efficacy phrases’ tends to focus on whether the recipe was considered ‘good’ or effective; but the reassurance that a recipe had been tried and/or was approved by a reliable person was also important in the process of establishing whether it was safe to use, as will be explored towards the end of this article. A connection between empirical testing and poisoning cannot, however, be ascertained from tag and efficacy phrases alone, which are often left out of recipes incorporating dangerous ingredients, but included in some mild recipes.

## Stupefactives

Recipes in manuscript collections are generally given without any comment on the medical theory behind them. However, comparing sleep recipes with advice concerning poisons found in vernacular medical guides suggests that printed texts had a significant influence on approaches to safety. In particular, herbals contain an abundance of information on the uses and dangers of various ingredients. Through studying them, lay practitioners acquired knowledge of a wide range of plants, their complexions, temperatures and virtues. Popular examples were Nicholas Culpeper’s *Astrologo-Physical Discourse of the Herbs of this Nation*, and works by William Turner and John Gerard.^33^

The advice found in these texts was underpinned by ancient principles of Hippocratic medicine. In the fifth century BC, a number of substances were classified as ‘refrigerants’, which were believed to have a soporific or pain-relieving effect on the body. Refrigerants worked by counteracting the heat that was causing pain or fever, and preventing sleep. These were further divided into mild (*mitigatives*) and strong (*stupefactives*). Wild lettuce was an example of an ingredient believed to be mildly sedative. In the early modern period, it was very popular as a remedy for sleep loss, often mixed with ‘woman’s milk’ (human breast milk). *Stupefactives*, on the other hand, did not simply provide relief, but had the power to numb parts of the body when applied locally, or to cause the whole body to fall into a lethargy (a deep, unresponsive sleep) when ingested. While *mitigatives* were considered safe, the use of *stupefactives* was often accompanied by dire warnings of the danger of death, and the advice to only use them in greatest need.^34^

*Stupefactives* were not strictly poisons, but dangerous drugs. Frederick Gibbs has shown how writers of poison treatises attempted to distinguish between *venenum*, which was fundamentally harmful to the human body, and strong medicines, which could harm the body through their qualities by introducing an excess of heat or cold.^35^ Commentators agreed that the coldness of *stupefactives* could be either therapeutic or deadly, depending on how they were administered and in what quantities. There were four plants in particular that were considered both potentially useful in sleep medicine, but also poisonous if used incorrectly: these were deadly nightshade, henbane, mandrake and the seeds or sap of the white (opium) poppy. While their deadly nature was well known, these plants had a history of use in surgery and medicine for pain relief and sedation stretching back to ancient times.^36^

Deadly nightshade, sometimes known by its Italian name *bella donna*, was much feared because the berries were fatal if ingested. Its native name was ‘dwale’, from the Scandinavian *dvale* meaning ‘stupor’. Herbalists warned against growing it in gardens where it could be accidentally eaten by children and expectant mothers. If it was cultivated it should be in a separate patch, fenced off. Jon Gerard, in a chapter on ‘sleepy nightshade’, described it as ‘a plant furious and deadly: for it bringeth such as have eaten thereof into a dead sleepe wherein many have died, as hath been often seen and prooved by experience both in England and else where’. However, its cooling properties were useful in external medicine: ‘The leaves heereof laid unto the temples cause sleepe, especially if they be imbibed or moistened in wine vineger. It easeth the intolerable paines of the head-ach proceeding of heate in furious agues, causing rest being applied as aforesaid.’^37^

Similarly *Hyoscyamus*, known as henbane in English, was believed to be of great help in headaches and fevers when in contact with the skin. Herbals advised making it into a plaster with woman’s milk, egg white and vinegar, or applying the oil to the temples to provide relief. The leaves should never be ingested, although the *Grete Herbal* (1526) suggested that the red and white seeds (never the black) were safe for ingestion in small doses.^38^ According to the Flemish botanist Rembert Dodoens, the smell helped a patient to sleep. One practice was to wash the feet with a concoction of the leaves seethed in water.^39^

Among physicians and herbalists mandrake had a reputation for acting as a strong narcotic even in the worst fevers, and could cause a sleep so deep that the patient would not feel the surgeon’s knife. The thirteenth-century text *On the Properties of Things*, an English edition of which was published in the late sixteenth century, advised that mandrake mixed with woman’s milk and applied to the temples ‘maketh to sléepe, yea, though it were in the most hot ague’. It could be ingested if necessary, but the writer advised great caution as an overdose would be fatal.^40^

The advice around these herbs was therefore very similar: they could be poisonous if ingested, and therefore should only be taken internally in the greatest need (in the case of mandrake and henbane), or not at all (in the case of nightshade); but they were safe to use in topical applications, having a therapeutic effect through smell or skin contact. This was because in Galenic medicine it was believed that the cooling powers of herbs would be absorbed by the body through the pores of the skin, helping to rebalance the humours. Poultices bound to the head would therefore affect the temperature of the brain, inducing sleep. Aromatics were important in this process, as smell was believed to be a vapour drawn directly into the brain through the nose.^41^

Recipes involving *stupefactives* in printed and manuscript sources were more likely to specify that they were intended for a patient who was sick, rather than simply having trouble sleeping at night. An example of how deadly nightshade was used in this context can be found in the diary of the merchant and astrologer Samuel Jeake. On a night when he was suffering from a fever, which kept him from sleep, he found relief by laying the leaves of the plant on his temples and forehead.^42^ There are also numerous examples in receipt books that suggest that external application was a popular way of exploiting the narcotic benefits of poisonous plants, while avoiding the danger associated with ingestion. Take, for instance, the following recipe using the ‘cooling’ properties of nightshade, houseleek and lettuce, found in Lady Ayscough’s receipt book:

For heat of the stomack burneing up to the head and hindering sleep Nightshade, sengreen [houseleek], & lettice, stamp them together, then putt itt into vessels till itt be cold then anoint ye temples very well with itt & soake a paire of Linnen cloath in the liquor & bind itt to the temples & forehead and itt will expell the heat of the stomack and cause rest.^43^

Another example is a receipt from a collection begun by Anne Brumwich in the early seventeenth century, entitled ‘A Medicine to pcure sleepe’. The instructions are to add a spoonful each of lettuce, henbane, white poppy, nightshade and oil of roses to a larger amount of rose water, breast milk and wine vinegar, which is then heated and absorbed into a rose cake, before being wrapped in a cloth with some grated nutmeg and laid warm over the patient’s forehead. This recipe does not mention fever, but the instruction to ‘let it lye foure and twenty houres’ suggests that this was a remedy intended for those ill enough to have taken to their beds.^44^ To relieve fever and help ‘weak’ patients to sleep, mandrake, henbane and nightshade, sometimes soaked in vinegar, were applied to the temples, either directly to the skin or in linen cloths or quilted bags; they were also made into pomanders for the patient to smell.^45^

## White Poppy and Diacodium

Whereas henbane, nightshade and mandrake were considered safe to use in general sleep medicine only when applied externally, white poppy was treated in a more versatile fashion. The plant *papaver somniferum* (‘sleep-inducing poppy’) was known in English texts as ‘white poppy’, to distinguish it from the red field poppy (*papaver rhæas*), but its colour is closer to pale purple. Dodoens described it as ‘betwixt white and red, changing towarde blacke, having black spottes'.^46^ Manuscript and printed texts show evidence of the home-grown white poppy in use in medicine, and also the use of opium, which was the dried sap of opium poppies cultivated in hot countries and imported. The imported opium was regarded as more powerful than products made from native poppies, but both were considered to be narcotics. In *Medicaments for the Poor*, translated by Nicholas Culpeper, the author explained that the seeds of white poppy were milder and could be safely eaten, but opium had a similar narcotic strength to mandrake and henbane.^47^

Whereas opium was seen as dangerous, white poppy can be found used in a similar way to a mild sedative such as lettuce. Banckes’s herbal describes the theory and use of poppy in sleep medicine: ‘The whyte Popy is colde and moyste and it is good to cause one to slepe … to provoke a slepe make a playster of [the seeds] with womans mylke and the whyte of an egge & lay it to ye temples’.^48^ Dodoens suggested that a decoction of the leaves and heads would bring about sleep when it was drunk, or used to bathe the head and hands.^49^

White poppy, alongside lettuce, is one of the most popular ingredients in sleep recipes in manuscript receipt books. The seeds were added to posset ale or wine, drunk mixed with almond milk and lettuce juice, and used in warm compresses applied to the temples.^50^ Jane Jackson’s collection contains a recipe very similar to the one suggested in Banckes’s herbal:

Another to cause a man to sleep Take white poppie and hengle seede and the white of an egge and womans milke medle all together/ and lay to his head and it shall make him soon to sleepe and helpe him./.^51^

A popular way of using white poppy in sleep medicine during this period was in the form of *diacodium*, a syrup made from the heads ground with sugar. An early reference to *diacodium* can be found in William Bullein’s *Dialogue Against the Fever Pestilence* (1564), in which he advises ‘Drinke your Diacodion at night, to reconcile slepe again’.^52^  *Diacodium* could be bought from the apothecary, but manuscript receipt books commonly contain recipes for *diacodium* or similar poppy syrups.^53^ One of the reasons for this may have been to ensure quality. In 1747, H. Pemberton wrote in his commentary upon the *London Dispensatory* that *diacodium* was ‘a medicine of such importance, that it ought to be made, as near as possible always to one and the same standard’.^54^ However, the household was sometimes seen as a better place for quality-control than the apothecary’s shop. Martha Bradley, in her mid-eighteenth-century popular household guide *The British Housewife*, advised families to make *diacodium* at home, as the apothecary often used coarse sugar or burned the syrup.^55^

Evidence from receipt books shows *diacodium* was taken on its own,^56^ or used as an additive to strengthen a recipe based on *mitigatives*. An example is this recipe from the early seventeenth century: ‘Take of white Lettice seede one ounze, and beate it in a morter, with a quantity of good white Sugar, until it do come to a moist Conserve, and if you can gett it putt thereto halfe a spoonefull of Diacodyum’. In other recipes it was added to juleps and taken in, or with, possets.^57^ Recipe titles suggest that it was used in cases where a chronic or severe condition was preventing sleep. In Philip Stanhope’s collection, there is a recipe for a sleeping cordial intended for someone suffering from ‘extremity of paine of the Goute’. This is a mixture of *diacodium* and cowslip syrup with other syrups and waters. The instructions are to take ‘two drachmes .. at the howre of sleepe’.^58^ A similar ‘lulling cordial’ combining *diacodium* with cowslip syrup, intended for women in the ‘lying in’ period after childbirth, can be found in a collection of recipes spanning the period 1688 to 1727, begun by a Mrs Meade. This is another occasion in which pain would likely have interfered with rest.^59^

Culpeper’s *Physical Directory* contained a discussion of which poppy-based medicines posed the greatest threat to the patient. Imported opium derived from poppies grown in hot countries was the coldest (and therefore the most potent). Syrup made from native white poppies was not as strong, but should still be used with caution—‘though they be no edge-tools, yet tis ill jesting with them’. It should not be given at the beginning of a fever, for instance. However, syrup made from red poppies was safe and effective to be used when the patient was suffering from fever or frenzy. This last claim was disputed: ‘Some are of opinion that these Poppies are the coldest of all other: beleeve them that list: I know no danger in this syrup, so it be taken with moderation ... and may safely be given in Frenzies, feavers and hot agues’.^60^ Most English sleep recipes called for white poppies, but red poppies were also supposed to be sleep inducing. Under an entry on ‘Corne Roses, or wild Popyes’ in the Freke family papers is written: ‘The Faculty of the wyld popye is like thatt of the other [white poppy] viz. to procure sleep’.^61^ The title of a recipe for a syrup made from red field poppies from the mid seventeenth century—‘a sirrup of Poppies to procure Sleep safely’—suggests that Culpeper’s belief in the mildness of this soporific was shared by others.^62^

## Opium

English poppies, therefore, were thought of as a relatively safe staple ingredient of sleep medicine; but what of the more potent sap that came from the heads of foreign poppies? Opium had been used as a sedative and pain-killer in topical applications, compresses, inhalations and internal medicines since the late medieval period. Medieval physicians advised that it should only be used in cases of persistent insomnia or unbearable pain.^63^ While opium, in the form of *laudanum*, was to become central to pain relief and sedation in later centuries, in sixteenth-century printed herbals it was treated much like henbane or mandrake; its usefulness as a narcotic was attributed to its coldness, and it was assumed that it worked in much the same way as any other *stupefactive—*through application to the skin, smell and (only in very small amounts) taken internally. Consistently, the advice was to apply it outwardly, and only when necessary. Take, for example, the section on opium in the *Grete Herbal*. Opium is described as cold in the fourth degree, and instructions are given ‘To cause a seke persone to slepe: Medle opium in womans mylke and put powdre of mandragora therto …[or] confyet opium with juce of an herbe called knotgrasse or corrigiole or with juce of henbane and make a playster therto’.^64^

Even applied externally, the advice was to use caution. Dodoens’ herbal contains warnings about the dangers of opium:

The use of Poppie is very evill and dangerous, and especially Opium, the which taken excessively, or to often applied upon the flesh outwardly, or otherwise without good consideration and advisement, it wyll cause a man to sleepe to muche, as though he had the Lethargie, which is the forgetful sicknesse, and bringeth foolish doting fansies, it corrupteth the sense and understanding, bringeth the Palsie, and in fine it killeth the body.^65^

Thomas Lupton’s book of secrets *A Thousand Notable Things* included a ‘marvellous water to provoke sleepe’ made from ground opium and garlic heads, used to anoint the temples and wrists: ‘And beware you minister nor use this, but uppon a great necessity,’ he added, ‘as in franticke persons’.^66^

The Swiss physician Théophile Bonet, whose *A Guide to the Practical Physician Shewing … the Truest and Safest Way of Curing All Diseases* was published in English towards the end of the seventeenth century, was of the opinion that the use of raw opium in topical applications was at best ineffective—at worst, it would overheat the brain. If this guide is typical, the advice to physicians in the seventeenth century was that opium in its pure form should very rarely be given, considering that *diacodium* and *laudanum* provided safer alternatives.^67^ This may explain why opium in its raw form is very rarely found as an ingredient in sleep recipes from seventeenth-century manuscript receipt books. I have only found one recipe that uses the traditional method. This is in an early seventeenth-century collection of recipes written by Anthony Lewis, owned by the Marquess of Dorset. The receipt is to help a weak patient to sleep: ‘take gume called Opaim the bignesse of a Pease [similar to a chickpea] & dissolve it in two spoone full of womans milke, or for want of it Rosewater, make it bloud-warme, & anoynt ye temples’.^68^ An anonymous collection from later in the century suggests swallowing a small amount of a combination of opium and henbane gum rolled in treacle (presumably to take away the bitter taste) to help the patient to sleep. The advice to ingest these strong substances, even in a small amount, is unusual. As there are no markings around the receipt there is no way of knowing whether this recipe was actually tried.^69^

## It ‘Mortyfyeth All the Wyttes’

Printed advice literature and handwritten recipes, therefore, both point to a culture of caution surrounding the use of poisons for the purposes of helping a weak or feverish patient to sleep. However, these same ingredients—mandrake, henbane, nightshade and opium—were treated differently when the purpose was to create a deep state of unconsciousness, rather than to help the body fall into a natural sleep. Mandrake, in particular, was believed to be useful in surgery because a patient under its effects lost all sense of pain. William Turner advised that if mandrake juice was either smelled or ingested the patient ‘shall fall into a forgetfull and slepishe drowsines’. However, there were dangers and side-effects. The surgeon Ambroise Paré wrote: ‘Mandrag taken in great quantity, either the root or fruit causeth great sleepines, sadness, resolution [dispersal or dissolution of the homours], and languishing of the body, so that after many scritches and gripings, the patient falls asleep in the same posture as he was in, just as if he were in a Lethargie.’^70^

As mandrake could kill, there was debate over whether ingestion was safe. John Pechey, on observing that the smell of the pulp and seeds was not noxious, concluded that that part of the plant was relatively innocuous.^71^ Turner advised that up to an ounce and a half of wine with mandrake juice mixed in could be given in preparation for surgery, or in cases where the patient was in great pain and having difficulty sleeping. However, as an overdose could be fatal, Turner devoted a large part of his discussion of the virtues of mandrake to instructions on ways to revive a patient when there were signs of poisoning. These included making the patient smell pepper or mustard, and applying vinegar and rose oil to the temples.^72^

Printed sources stressed the potency of *stupefactives* and the powerful effects of miniscule amounts. The *Grete Herbal* informed the reader that a single grain of opium taken in the body ‘astonyeth and mortyfyeth all the wyttes of man in suche maner that he feleth no payne & causeth hym to slepe’. William Langham gave the dose to cause unconsciousness as the size of a single corn of wheat: ‘To sleepe while ye surgian do his office’ the patient should drink half a dram of opium mixed into a pound of wine.^73^ An alternative, that would avoid ingestion, was to create a powerful inhalant. The Italian surgeon Giovanni da Vigo, whose works were published in English in 1543, recorded that some gave the patient a sponge containing opium to smell, in order to render the patient unconscious before amputation—‘They enterpryse a daungerouse busyness’, he added.^74^  *The Treasury of Health* (1553) suggested soaking a sponge in the juice of opium, henbane, poppy, mandrake, mulberries, hemlock and lettuce, and holding this to the patient’s nose. This would cause the patient to sleep until he was revived using a sponge soaked in vinegar.^75^

Evidence of the use of *stupefactives* in this way in English domestic medicine is scarce. However, sleeping potions did form part of the secrets tradition with which household practitioners were experimenting in medieval and early modern Europe. Meredith Ray has found an opium-based surgical drink in a book of ‘experiments’ compiled by the Italian noblewoman Caterina Sforza in the late fifteenth century.^76^ In seventeenth-century English manuscripts, there are a small number of examples of drinks intended to stupefy the patient, which were used either in preparation for surgery or to incapacitate a person who had become frantic. Here, the printed and the manuscript sources diverge. While mandrake as an ingredient in sleeping potions appears in famous English plays, such as *The Jew of Malta*, it was not easily obtainable in England, and does not feature in these recipes.^77^

The basis of the drugs found in English receipt books is animal gall mixed with alcohol.^78^ A very small number of these also contain poisonous plants. One recipe in Jane Jackson’s collection, with the unsettling title ‘to make a drinke to cause a man to sleepe till hee bee ript’, involved mixing pig’s gall with the juice of hemlock, henbane, wild neep (bryony), lettuce, opium poppy and eysel (wine vinegar). This was then added to a little wine and drunk by the patient, who should be seated near a fire. The recipe promised that, once sleep occurred, ‘then mayst thou surely carve him’. When the operation was completed, the patient was revived by washing his temples and the wound with salt and vinegar.^79^

This recipe is recognisable as *dwale*, a narcotic drink^80^ found in English remedy books widely circulated from the late fourteenth century. It can also be found in Lupton’s *A Thousand Notable Things*, and in the physician and surgeon Thomas Bonham’s collection of recipes for use in surgical practice, published in 1630. Both sources refer to the age of the recipe as a basis for its authority. Lupton gives further insight into practices surrounding safe administration of the drug: ‘Use it warely and prove it advisedly: if you begyn with a lytle quantitie you maye increase it when you wyll: but if you geve too much at once, you can not diminish it when you lyst’.^81^

Linda Voigts and Robert Hudson, in their essay on *dwale* in the medieval period, note that this was a peculiarly English recipe. Firstly, it did not include mandrake, secondly, continental texts on surgery tended to prefer the soporific sponge described in da Vigo’s treatise. While many of the ingredients can be explained in terms of Galenic humoral theory, Voigts and Hudson came to the conclusion that the recipe was arrived at empirically, rather than copied between manuscripts, and represents actual medical practice in medieval England. However, ancient and medieval theories about the safe use of *stupefactives* can clearly be seen influencing the ingredients and instructions written into the recipe. It was thought that henbane and white poppy were more safely used in combination than separately, and that vinegar was a powerful antidote to narcotic poisons. Keeping the patient near the fire was another preventative measure against the danger of death, the heat from the fire counteracting the cold effects of the drug upon the humours.^82^ The recipe, therefore, represents both experiential knowledge, in terms of expertise acquired through surgical practice and familiarity with native English plants, and theoretical knowledge, in terms of a Galenic framework for approaching poisons. It was this theoretical framework, which advocated mixing poisonous ingredients, untreated, with ingredients thought to counteract the poison, that was vigorously rejected by Paracelsus and his followers.

## Preparing Laudanum

Casting Hippocratic medicine as unclean, malignant and deadly, Paracelsus and his followers portrayed chymical medicines as powerful and pleasant—an attractive alternative to the violent and ineffective methods of trying to balance the humours through blood-letting, purges and mixing poisons with ‘corrective’ ingredients.^83^ A recent paper by Georgina Hedesan has greatly expanded our understanding of Paracelsus’ theory of poisons. He regarded any poison that had not been alchemically treated as harmful to the body, even in small doses, and was highly critical of how dangerous ingredients were added, untreated, to remedies in traditional medicine. Potentially deadly substances, however, could form the basis of powerful remedies because every substance contained within it both toxin and cure (or *arcana*). The aim of alchemical medicine was to extract the *arcana* by separating the substance into its constituent parts of salt, mercury and sulphur. The vapours of mercury or sulphur were poisonous, but the salt was generally good for health.^84^

The Paracelsian tradition placed a high value on sedative medicine, emphasising the central role of sleep in the restoration of health: ‘Sleep is the *Arcanum* in medicine,’ wrote the sixteenth-century alchemist Oswald Croll, ‘far more worth than gemms or precious stones’.^85^  *Laudanum* was developed through attempts to produce a narcotic more powerful and effective than traditional remedies by following Paracelsus’ radical new theory of how to turn poisons into medicines. Some very early recipes for *laudanum* did not contain opium, but did contain henbane gum.^86^ It may have been the case that, at this stage (late sixteenth century), opium was regarded as being interchangeable with henbane because both were *stupefactives*. This would suggest that it was the application of the processes of alchemical transformation to the traditional ingredients of sleep medicine, rather than the presence of opium, that was thought of as *laudanum’*s essential characteristic. The drug sent to Lord Burghley may not have been opium-based.

*Laudanum* promised a new thing: a powerful internal medicine that could be used in cases where fever and pain were interfering with rest. However, this challenged the centuries-old belief that potent medicines should only be ingested in ‘desperate diseases’. In 1618, the astrologer and medical practitioner Thomas Bretnor published a translation of a French work on how to prepare *laudanum* ‘for the comfort and ease of all such persons as are inwardly afflicted with any extreme griefe, or languishing paine, especially such as deprive the body of all natural rest’. In a preface to the reader, he explained that his primary motivation for publishing the pamphlet was that both physicians and lay people had serious reservations about the safety of *laudanum*:

not onely the rude multitude and men of some judgement through vaine delusion and superstitious feare, but many Physitions themselves through a *Galenicall* perswasion, make no small question and scruple whether *Opium* may bee taken inwardly or not; Nay, I have knowen men of good discretion so farre infatuated by conceipt and heare say as they have rather chosen to indure intollerable paines then they would take three poore graines of well prepared *Laudanum*

Bretnor believed that distrust in *laudanum* was fostered by wide-spread ignorance concerning alchemical processes: ‘I cannot greatly blame them,’ he continued, ‘for the naked truth is, many modern Physitions know not well what to make of it … I onely wish you to have great regard to its preparation: for as in most Physicall Drugges there remaineth some bad qualitie or other which needeth correction’.^87^

The refining process by which the *arcana* was extracted was known amongst alchemists as the art of *spagyria*.^88^ Bretnor’s pamphlet, originally written by the alchemical physician Angelus Sala, discussed the properties of raw opium and explained how the *spagyrist* turned it into *laudanum*. Some physicians, from the Paracelsian tradition and more broadly, questioned whether opium had been correctly classified as cold in complexion, and therefore whether its observed narcotic and deadly effects could be explained using the traditional Galenic framework.^89^ However, when it came to views of poisoning, the question was not of primary importance because Paracelsians attributed the lethal potential of opium to the sulphur contained within the raw product.

After outlining the various ailments for which opium could be used externally, and the various arguments over the safety of ingestion, Sala wrote ‘it is not my counsel that any Physition should use it crude in this maner, seeing we have Art and meanes to prepare it otherwise’. He went on to describe the various means of removing the noxious sulphur, which included infusion with aromatics or the juice of quinces, baking, heating in a frying pan to evaporate off the sulphur, and dissolving in liquor; the *quintessence* (pure essence) of opium was then extracted by mixing with the juice of quinces, vinegar or liquor.^90^

There are a number of examples of *laudanum* in use as a sleep remedy recorded in manuscript receipt books.^91^ However, throughout the seventeenth century, concerns were raised that many physicians continued to be ignorant of the proper way of making and administering it. Thomas Willis wrote, disparagingly, of the many novel medicines that had come into use under the name of *Laudanum* or *Nepenthes*: ‘among our Countrymen there is not only a company but a swarm rather of false Chymists and Quacksalvers; every one of which boasts of his peculiar *Laudanum*, which they rashly give in every Disease and Condition’.^92^

This may be why *laudanum* does not appear so commonly in sleep recipes as *diacodium*, which was often seen by health-care advisers as a safer substitute. Bradley claimed that her recipe for home-made *diacodium* was ‘much safer and better than *Laudanum*’.^93^ Gideon Harvey considered *diacodium* to be an opiate, with similar effects to *laudanum*—‘though in a milder degree, all do assent *Diacodium* to be’.^94^ Bonet’s guide suggests that, towards the end of the seventeenth century, a hierarchy of opiate medicines had developed, with the strongest and most dangerous being reserved for intractable cases of pain and fever. He directed that drugs should only be given at all when pain and want of sleep was causing a serious deterioration in the patient, and only when milder remedies had failed. *Diacodium* was the safest, and should be tried first, followed by *laudanum* only if necessary: ‘For it is not good to ascend to *Laudanum*, unless when through the vehemence of Symptoms Diacodiats will not do’.^95^

Doubts concerning the standards of ready-made *laudanums* may also be why some household receipt collections contain their own recipes for preparing it. Alisha Rankin has found that German gentlewomen undertook laborious tasks in preparing medicines, and went to some trouble to gain exact instructions on the methods to be used to ensure the quality and safety of the medicines they created.^96^ Recipes for *laudanum* suggest a similar ambition among lay practitioners in England. These required knowledge and skill in pharmaceuticals, in the form of the methods of infusing, heating and dissolving described in Bretnor’s pamphlet. In 1712, Elizabeth Freke’s sister sent her a recipe for *laudanum* she had obtained from Lady Powell, which involved infusing opium with saffron and sack wine in an earthen pot. The instructions state that it ‘will be a great while’ before the infusion process is complete.^97^ A recipe from Mrs Meade’s collection entitled ‘To make Laudanum van: Helmetts way’ instructs the reader to heat opium, mixed into quince juice, in a pot near a fire for three weeks before evaporating off the liquid.^98^ An anonymous cookery book includes a recipe ‘to make Liquid laudanum’ that involves infusing opium with saffron, cinnamon and powdered cloves in a glass bottle, and heating it for ‘dayes upon soe much fire as may keep ye water in a temperate & constant heat’.^99^

In the records of the Boyle family there are two recipes for *laudanum*. One of them, made through a process of fermentation and distillation, contains a number of sensual clues for the maker to know whether the process is being followed successfully. When the mixture is first heated it ‘will conceive a ferment and exhale a very strong stink’. The mixture is then left to ferment, which creates ‘plentifull feaces’. At the end of the process of fermentation, filtering and distillation, ‘it will be a sweet smelling laudanum’.^100^ Smell was an important way of detecting poison in early modern medicine. A major skill in pharmaceuticals was being able to evaluate a substance by means of smell, sight, taste and touch.^101^

Robert Boyle took a great interest in the advancement of chymical medicine, and in *laudanum* in particular, exchanging letters with physicians concerning the effectiveness of the drug in treating various illnesses. In 1674, he published a paper for the Royal Society on methods of preparing *laudanum*, based on instructions he claimed to have been given by the son of Jan Baptiste van Helmont. The method is similar to that contained in the Boyle family receipt book.^102^ While other lay practitioners had less interest in the advancement of natural philosophy, their attempts to make *laudanum* to find relief from their own ailments were no less ambitious.

Recipes for *laudanum* in receipt books therefore reflect the experimental nature of *laudanum* at this stage of its development. There was no standardised recipe; rather *laudanum* referred to opium (or, occasionally, henbane) that had been processed using pharmaceutical methods designed to remove the poisonous element. It is possible that housewives created their own versions of *laudanum* to save on costs (as Bretnor advised physicians to do).^103^ However, the amount of time and effort involved suggests that this was part of the culture of trials and testing by which individuals sought to determine for themselves which medicines were safe and effective. It is to this issue of personal experience, and its importance in establishing trust in *laudanum*, that I turn in the next section.

## Testing Laudanum

Unlike traditional sleep remedies, *laudanum* had not been tested by centuries of use. As Bretnor’s treatise and the letter from Talbot examined at the beginning of this article suggest, chymical medicines were regarded by some as a dangerous novelty. Personal recommendation was therefore particularly important if *laudanum* was to become accepted outside the realm of alchemical experiment. Letters such as the one from Lord Talbot provide an insight into the kind of anecdotal evidence on which friends and family relied when trying a new remedy. Such experience appears to have been valued above the advice of medical practitioners. In his letter Talbot assures Burley that, although the apothecary advised that no more than three grains of *laudanum* should be taken at once, his wife had taken four with no ill effects.^104^

Elizabeth Freke’s sister, Judith Austen, included in the letter, which contained the recipe for *laudanum*, a lengthy description of the various illnesses (suffered by herself, neighbours and family members) that had been helped by taking the drug, together with warnings about side-effects and suggested dosage. ‘All these informations I give my deerest sister,’ she wrote, ‘thatt you may see itt is a safe thinge and nott be affraid of itt otherwise then to have itt carefully droptt.’ The letter suggests that, by the eighteenth century, *laudanum* users were taking far more than the cautious doses recommended in printed texts and manuscript receipt books, as sufferers from chronic conditions became addicted. Both Austen and her sister were affected by bouts of colic. Austen describes how she has been compelled by acute pain to increase the dose in order to regain some quality of life. She began with twenty drops, but was obliged to enlarge this to twenty five with frequent use. She advises her sister that, to be effective in severe cases, this dose must be doubled, although ‘itt sometimes flyes to the head, as perhaps you may find, and to the frightning of you’. Austen adds that her daughter (also suffering from colic) takes *laudanum* frequently to help her sleep, and that a neighbour takes thirty drops twice a day. Lady Powell (the original donor of the recipe) has taken fifty drops a day for the past several years, having turned to *laudanum* after other means of relief failed—‘she had bin dead many years since butt for itt—shee haveing tryed all the conciderable phisitions in London and by them brought to a skellitton in weakness and to noe effectt’.^105^

Austen herself was positive about the safety of *laudanum* use on a long-term basis. Others were unconvinced, and remained suspicious of the drug. In an entry in her diary in 1704, Lady Cowper recorded that the Duchess of Buckingham had such trouble sleeping that she took 150 drops of *laudanum* each night (a claim probably exaggerated by rumour). Cowper seems to see this as a sign of desperation: ‘I need somewhat to make me sleep’, she adds, ‘... but neither Cares nor Fears have yet reduced me to such Remedys’.^106^

## Conclusion

Placing *laudanum* in the context of sixteenth- and seventeenth-century approaches to dangerous medicines enables us to assess its significance. Traditional advice literature strongly discouraged ingestion of strong narcotics, except in the very exceptional circumstances of surgery. The great majority of sleep recipes found in manuscript receipt books follow this advice. Yet the letters sent to Lord Burghley and Elizabeth Freke express enthusiasm for a novel medicine involving potentially lethal ingredients designed to be swallowed, contrary to this advice. There are two possible explanations for this. One is that the herbal compresses and possets were not very effective in cases of chronic conditions such as colic. The description of cases in the letter to Elizabeth Freke suggests this.

Another possibility is that lay practitioners and patients were more concerned with evidence of the effects of a medicine, than with whether it was Galenic or Paracelsian. There is a high level of theory underpinning the recipes included in manuscript receipt books, but how well this theory was understood by those who collected and used them is not so clear. Whereas for Bretnor, understanding first principles was essential for preparing *laudanum* properly, the two letters examined in this article suggest that, in a lay context, personal recommendations and testing were more important considerations when assessing whether a particular preparation was safe for use. Lay people undertook to advise on dosage, based on personal experience, or that of a close relative or neighbour, sometimes in contradiction to professional medical advice.

This provides further insight into the importance of the household as a centre for quality-control and testing. It would be wrong to see ambitious attempts to recreate and test new medicines in the household as foolhardy. In the context of a medical market in which medicaments were variable in quality and contents, knowledge of ingredients and processes from first-hand experience, or that of a trusted friend, were highly valued as a guarantee of safety, as well as of success. However, with evidence of people resorting to high dosages of *laudanum* as rest remained elusive, and concerns about the side-effects of the drug, this success was ambivalent.

## Funding

This research was funded in whole, or in part, by The Wellcome Trust [Grant number 109069/Z/15/Z].

## Footnotes

1 Lord Talbot to Lord Burghley, March 30, 1558, No. 39, Lansdowne MS 57, British Library, London (henceforth BL).

2 Modern sleep research defines sleep as a spontaneous and easily reversible state, which erects a perceptual wall between the conscious mind of the sleeper and the outside world: Matthew Wolf-Meyer, ‘The Nature of Sleep’, *Comparative Studies in Society and History*, 2011, *53*, 945–70, at 946–47. Early modern concepts of sleep were broader than this, and included states such as coma and lethargy: Margaret Simon and Nancy Simpson-Younger, ‘Introduction: Forming Sleep’, in Simon and Simpson-Younger, eds, *Forming Sleep: Representing Consciousness in the English Renaissance* (Pennsylvania Park, PA: Pennsylvania State University Press, 2020), 5–6.

3 A. Roger Ekirch, *At Day’s Close: A History of Nighttime* (London: Phoenix, 2006), ch. 11; Janine Rivière, *Dreams in Early Modern England: Visions of the Night* (London: Routledge, 2017), 137–39.

4 Sandra Cavallo and Tessa Storey, *Healthy Living in Late Renaissance Italy* (Oxford: Oxford University Press, 2014), ch. 4; Sasha Handley, *Sleep in Early Modern England* (London: Yale University Press, 2016), ch. 2.

5 Hannah Newton, ‘“She Sleeps Well and Eats an Egg”: Convalescent Care in Early Modern England’, in Sandra Cavallo and Tessa Storey, eds, *Conserving Health in Early Modern Culture: Bodies and Environments in Italy and England* (Manchester: Manchester University Press, 2017), 104–32, at 111–13.

6 Sasha Handley, ‘Sleep-piety and Healthy Sleep in Early Modern English Households’, in *Ibid*, 185–209, at 200–03.

7 Sasha Handley, ‘Accounting for Sleep Loss in Early Modern England’, *Interface Focus*, 2020, *10* [https://doi.org/10.1098/rsfs.2019.0087], sect. 6, para. 5. Tanya Pollard has drawn attention to the relationship between sleeping draughts and poisons as a theme in Shakespeare’s plays. She suggests that innovations in alchemical medicine added to the fear and uncertainty surrounding sleep medicine: Tanya Pollard, ‘“A Thing Like Death”: Sleeping Potions and Poisons in Romeo and Juliet and Antony and Cleopatra’, *Renaissance Drama*, 2003, *32*, 95–121.

8 Andrew Cunningham, ‘Introduction: Deadly Medicine’, and Helen King, ‘“First Behead Your Viper”: Acquiring Knowledge in Galen’s Poison Stories’, in Ole Peter Grell, Andrew Cunningham and Jon Arrizabalaga, eds, *‘It All Depends on the Dose’: Poisons and Medicines in European History* (e-book, Routledge, 2018), ch. 2, paras. 3–9. For an older study, which explores the subject from the standpoint of modern toxicology, see John Timbrell, *The Poison Paradox: Chemicals as Friends and Foes* (Oxford: Oxford University Press, 2005).

9 Frederick W. Gibbs, *Poison, Medicine, and Disease in Late Medieval and Early Modern Europe* (e-book, Routledge, 2018), ch. 6, subheading 3, paras. 4–9.

10 Elaine Leong, *Recipes and Everyday Knowledge: Medicine, Science and the Household in Early Modern England* (Chicago, IL: University of Chicago Press, 2018); Wendy Wall, *Recipes for Thought: Knowledge and Taste in the Early Modern Kitchen* (Philadelphia, PA: University of Philadelphia Press, 2016), esp. ch. 5; Alisha Rankin, *Panaceia’s Daughters: Noblewomen as Healers in Early Modern Germany* (Chicago, IL: The University of Chicago Press, 2013).

11 William Eamon, *Science and the Secrets of Nature: Books of Secrets in Medieval and Early Modern Culture* (Princeton, NJ: Princeton University Press, 1994).

12 Pamela H. Smith, ‘What is a Secret? Secrets and Craft Knowledge in Early Modern Europe’, in Alisha Rankin and Elaine Leong, eds, *Secrets and Knowledge in Medicine and Science, 1500-1800*, 2nd edn (London: Routledge, 2016), 47–68; Hannah Bower, ‘The Problem with Practicality: Rethinking Late Medieval English Medical Recipes, 1375-1500’ (DPhil thesis, University of Oxford, 2018).

13 See references at nt. 10.

14 Elizabeth Spiller, *Seventeenth-Century English Recipe Books: Cooking, Physic and Chircurgery in the Works of Elizabeth Talbot Grey and Aletheia Talbot Howard* (London: Routledge, 2008), x.

15 Seth Stein LeJacq, ‘The Bounds of Domestic Healing: Medical Recipes, Storytelling and Surgery in Early Modern England’, *Social History of Medicine*, 2013, *26*, 451–68.

16 Alisha Rankin, ‘Gender, Poison, and Antidotes in Early Modern Europe’, in Cunningham and Grell, eds, *It All Depends*, ch. 8; Meredith K. Ray, ‘Prescriptions for Women: Alchemy, Medicine and the Renaissance Querelle Des Femmes’, in Anke Gilleir, Alicia Montoya and Suzan van Dijk, eds, *Women Writing Back/ Writing Women Back: Transnational Perspectives from the Late Middle Ages to the Dawn of the Modern Era* (Leiden: Boston, 2010), 135–61; Elaine Leong, ‘Making Medicines in the Early Modern Household’, *Bulletin of the History of Medicine*, 2008, *82*, 145–68.

17 Over 80 manuscript receipt books were consulted during research for this article. Much of this was made possible by the provision of online digital collections by the Folger Shakespeare Library [https://luna.folger.edu/luna/servlet/] and the Wellcome Library [https://wellcomelibrary.org/collections/digital-collections/recipe-books/].

18 Alix Cooper, ‘Home and Households’, in Katharine Park, ed, *The Cambridge History of Science: vol. 3 Early Modern Science* (Cambridge: Cambridge University Press, 2006), 224–37, at 227.

19 These involve placing animal parts, plants or inscriptions upon the breast of the patient: Thomas Sheppey, ‘A Book of Choice Receipts … ca. 1675’, v.a.452, Folger Shakespeare Library, Washington (henceforth FSL), 318; Jane Jackson, ‘A very shorte and compendious Methode of Physicke and Chirurgery, 1642’, MS 373, Wellcome Library, London (henceforth WL), fo. 98r.

20 'The Lady Ayscough Booke Anno Domini 1692', MS 1026, WL, 127.

21 Elizabeth Okeover & Others, c. 1675-1725, MS 3712, WL, fo. 101v.

22 Philip Stanhope, ‘A Booke of Severall Receipts for Severall Infirmities’, 2 vols, vol. 1, MS 761, WL, fo. 7r.

23 Sir Hugh Platt, ‘Collection of Medical Receipts’, Sloane MS 2203, BL, fo. 62r.

24 'A Book of Phisick. Made June 1710', MS 1320, WL, 107, 109; Boyle Family, c. 1675-1710, MS 1340, WL, fo. 121v. A grain was the smallest English unit of weight, equivalent to a corn of dried wheat: see entry for ‘grain, n1’ meaning 8 in the *Oxford English Dictionary Online* [www.oed.com].

25 'My Lady Rennelaghs Choice Receipts', MS Sloane 1367, BL, fo. 9v.

26 Elizabeth Jacobs & Others, 1654 – c. 1685, MS 3009, WL, 57.

27 Anne Lovelace, ‘Recipes Medical and Culinary’, Add. MS 34722, BL, fo. 41r.

28 Leong, *Recipes and Everyday Knowledge*, chs. 3–4.

29 Lady Frances Catchmay, ‘A Booke of Medicens’, c. 1625, MS 184A, WL, fo. 20r; English Receipt-Book: 16th Century, MS 634, WL, 138; Anon, ‘A Book of Receipts Which Was Given Me By Several Men … ca. 1625-1700’, v.a.361-362, FSL, fo. 40r; FSL v.a.452 (Thomas Sheppey), 318–19.

30 The empirical basis of efficacy phrases is difficult to establish, as ‘proved’ in the medieval period could also refer to the method of scholastic proof demanded in learned medical circles: Claire Jones, ‘Formula and Formulation: “Efficacy Phrases” in Medieval English Medical Manuscripts’, *Neuphilologische Mitteilungen*, 1998, *99*, 199–209; Sara Pennell does not consider the phrase *proabtum est* as sufficient in itself to indicate that a recipe had been tried, as the phrase may have been copied from a printed text: Sara Pennell, ‘Perfecting Practice? Women, Manuscript Recipes and Knowledge in Early Modern England’, in Victoria E. Burke and Jonathan Gibson, eds, *Early Modern Women’s Manuscript Writing* (London: Routledge, 2004), 237–58, at 255, nt. 28. Rebecca Bushnell points out that books of secrets often claimed that their fantastic cures had been ‘proved’—claims which began to be doubted as the new science of empiricism took hold: Rebecca Bushnell, *Green Desire: Imagining Early Modern Gardens* (Ithaca, NY: Cornell University Press, 2003), 177–81. Wendy Wall, while she acknowledges that sometimes the phrase *probatum est* was applied to cures that were highly unlikely, argues that it is counter-intuitive to dismiss it, given the abundance of other evidence of testing within receipt books: Wall, *Recipes for Thought*, 212–18, ch. 5 nt. 10. The most significant recent contribution to the debate is that of Elaine Leong, who has found evidence directly linking the phrase to personal experience of either the compiler or the donor: Leong, *Recipes and Everyday Knowledge*, 84–86.

31 FSL v.a.361-362 (Anon, ‘A Book of Receipts’), fo. 40r; FSL v.a.452 (Thomas Sheppey), 319.

32 Rankin, *Panaceia’s Daughters*, 39.

33 Elaine Leong, ‘“Herbals she Peruseth”: Reading Medicine in Early Modern England’, *Renaissance Studies*, 2014, *28*, 556–78.

34 G. Kasten Tallmadge, ‘Some Anesthetics of Antiquity’, *Journal of the History of Medicine and Allied Sciences*, 1946, *1*, 515–20. Faye Marie Getz, *Healing and Society in Medieval England: A Middle English Translation of the Pharmaceutical Writings of Gilbertus Anglicus* (London: University of Wisconsin Press, 1991), 38–39. Gilbertus Anglicus was writing in the context of cures for eye ache, which have many similarities with cures for headache and sleeplessness: ‘Strong repercussives are called *stupefactives* because they stupefy a part through their cold and so they make it lose its ache and its feeling. And because of their great violence, you shall not take them in the ache of the eyes nor in any other ache unless the ache is very violent and dangerous’. For examples of lettuce mixed with breast milk see FSL v.a.452 (Thomas Sheppey), 318–19. For an introduction to sleep in English medicine see Karl H. Dannenfeldt, ‘Sleep: Theory and Practice in the Late Renaissance’, *The Journal of the History of Medicine and the Allied Sciences*, 1986, *41*, 415–41.

35 Frederick W. Gibbs, ‘Specific Form and Poisonous Properties: Understanding Poison in the Fifteenth Century’, *Preternature: Critical and Historical Studies on the Preternatural*, 2013, *2*, 19–46, esp. 22–28.

36 Ioanna A. Ramoutsaki, Helen Askitopoulou, Eleni Konsolaki, ‘Pain Relief and Sedation in Roman and Byzantine Texts: Mandragoras Officinarum, Hyoscyamos Niger and Atropa Belladona’, *International Congress Series: The History of Anesthesia*, 2002, *1242*, 43–50; John Scarborough, ‘The Opium Poppy in Hellenistic and Roman Medicine’, in Roy Porter, ed, *Drugs and Narcotics in History* (Cambridge: Cambridge University Press, 1995), 4–23.

37 John Gerard, *The Herball or Generall Historie of Plantes* (London, 1633), 340–41; Rembert Dodoens, *A Newe Herbal, or Historie of Plantes*, trans. Henry Lyte (London, 1578), 447.

38 Anon, *The Grete Herball which Geveth Parfyt Knowledge and Understanding of All Maner of Herbes* (London, 1526), sig. N(v); Nicholas Culpeper, *The English Physitian Enlarged … Being An Astrologo-physical Discourse of the Vulgar Herbs of this Nation* (London, 1653), 124–25.

39 Dodoens, *A Newe Herbal*, 450.

40 Stephen Batman, *Batman Upon Bartholome His Booke De Proprietatibus Rerum*, trans. John Trevisa (London, 1582), fo. 304r-v.

41 C. M. Woolgar, *The Senses in Late Medieval England* (London: Yale University Press, 2006), 58, 126.

42 Michael Hunter and Annabel Gregory, eds, *An Astrological Diary of the Seventeenth Century: Samuel Jeake of Rye, 1652-1699* (Oxford: Clarendon Press, 1988), 140.

43 WL MS 1026 (Lady Ayscough), 189–90.

44 'Mrs Anne Brumwich Her Booke of Receipts or Medicines ffor Severall Sores and other Infermitie', c. 1625–1700, MS 160, WL, 54. On the concepts of a ‘weak’ patient and taking to bed see Hannah Newton, *Misery to Mirth: Recovery from Illness in Early Modern England* (Oxford: Oxford University Press, 2018), 68–71, at 195–98.

45 Anthony Lewis, ‘Receipts in Cookery, Confectionery, Gardening, Medicine’, owned by the Lady Marquess Dorset, ca. 1606/7, MS Sloane 556, BL, fo. 39r; Pharmaceutical and Cookery Recipes, ca. 1675, v.a.21, FSL, 18, 55; FSL v.a.452 (Thomas Sheppey), 318; Arthur Corbett, Collection of Medical Receipts, Middle 17th Century, MS 212, WL, fo. 84r; WL MS 373 (Jane Jackson), fo. 37r; Receipt Book, Early 17th Century, MS 8086, WL, 151.

46 Dodoens, *A Newe Herbal*, 432.

47 Jean Prevost, *Medicaments for the Poor*, trans. Nicholas Culpeper (London, 1656), 263–64.

48 Anon, *Here Begynnyth a New Mater, the Whiche Sheweth and Treateth of ye Vertues [and] Proprytes of Herbes* (London: Rycharde Banckes [printer], 1525), sig. Gii(r).

49 Dodoens, *A Newe Herbal*, 432.

50 See for example Book of Receipts, A, c. 1650–1739, MS 144, WL, fo. 53r; WL MS 373 (Jane Jackson), fo. 47r; WL MS 1026 (Lady Ayscough), 241; WL MS 8086 (Receipt Book), 152; Mrs Corlyon, ‘A Booke of Such Medicines As Have Been Approved … ca. 1606’, v.a.388, FSL, 18-19; Cookbook of Constance Hall, 1672, v.a.20, FSL, fo. 18r; Medical Recipes, Compiled ca. 1625, x.d.469, FSL, 23.

51 WL MS 373 (Jane Jackson), fo. 91v.

52 William Bullein, *A Dialogue Both Pleasant and Piety-full, Against the Fever Pestilence* (London, 1564), fo. 37v.

53 Cookery and Medical Receipts by Margaret Barker, 1672, MS Sloane 2485, BL, fo. 14v; Katherine Brown, Medicinal and Cookery Recipes, ca. 1650-1662, v.a.397, FSL, fo. 44r.

54
*The Dispensatory of the Royal College of Physicians, London*, trans. and comment. H. Pemberton (Dublin, 1747), 218–19.

55 Martha Bradley, *The British Housewife; Or, the Cook, Housekeeper and Gardiner’s Companion* (London, [1770?]), 384–85.

56 WL MS 1320 (Book of Phisick), 107.

57 FSL v.a.388 (Mrs Corlyon), 18–19; John and Joan Gibson, Medical Recipe Book, 1632–1717, MS 311, WL, 30; 'Johanna Saint John Her Booke, 1680', MS 4338, WL, fo. 138r; English Medical Notebook, 17th Century, MS 6812, WL, fo. 41r.

58 WL MS 762 (Philip Stanhope, vol. 2), fo. 293r.

59 WL MS 3500 (Mrs Meade & others), fo. 30v.

60 Nicholas Culpeper, *A Physicall Directory, or, A Translation of the London Dispensatory Made by the Colledge of Physicians in London* (London, 1649), 112–13, 131.

61 Freke Papers, vol. I, Commonplace Book of Elizabeth Freke, Add. MS 45718, BL, 275.

62 English Recipe Book, Seventeenth Century, MS 7391, WL, 7.

63 Luke DeMaitre, *Medieval Medicine: The Art of Healing, from Head to Toe* (Santa Barbara, CA: Praeger, 2013), 125.

64 Anon, *The Grete Herball*, sig. S(v) – Si(r). For other examples of opium used externally, see Humphrey Llwyd (trans. and exp.), *The Treasury of Healthe* (London, 1553), sig. C.iii(v) – C.iv(v); Philip Barrough, *The Method of Phisick: Containing the Causes, Signes and Cures of Inward Diseases* (London, 1583), [8]; William Langham, *The Garden of Health Conteyning the Sundry Rare and Hidden Vertues and Properties of all Kindes of Simples and Plants* (London, 1597), 454; Ralph Williams, *Physical Rarities Containing the Most Choice Receipts of Physick and Chyrurgerie* (London, 1652), 136.

65 Dodoens, *A Newe Herbal*, 433.

66 Thomas Lupton, *A Thousand Notable Things, of Sundry Sortes* (London, 1579), 109.

67 Théophile Bonet, *A Guide to the Practical Physician Shewing … the Truest and Safest Way of Curing All Diseases*, trans. anon (London, 1686), 751–52.

68 BL Sloane 556 (Anthony Lewis), fo. 39r.

69 FSL v.a.21 (Pharmaceutical and Cookery), 7.

70 William Turner, *The First and Seconde Partes of the Herbal of William Turner Doctor of Phisick* (Cologne, 1568), 47; Ambroise Paré, *The Works of that Famous Chirurgion Ambrose Parey*, trans. Thomas Johnson (London, 1665), 518. See also Pierre Boaistuau, *Certaine Secrete Wonders of Nature Containing a Description of Sundry Strange Things*, trans. E. Fenton (London, 1569), 73; Batman, *Batman Upon Bartholome*, fo. 304r-v.

71 John Pechey, *The Compleat Herbal of Physical Plants* (London, 1694), 297.

72 Turner, *Herbal of William Turner*, 47.

73 Anon, *The Grete Herball*, sig. S(v) – S.i(r); Langham, *The Garden of Health*, 454.

74 Giovanni da Vigo, *The Most Excellent Workes of Chirurgerye*, trans. Bartholomew Traheron (London, 1543), sig. C.lix(v).

75 Llwyd, *Treasury of Healthe*, sig. C.iii(v) – C.iv(v).

76 Meredith K. Ray, ‘Experiments with Alchemy: Caterina Sforza in Early Modern Scientific Culture’, in Kathleen P. Long, ed, *Gender and Scientific Discourse in Early Modern Culture* (Farnham: Ashgate, 2010), 139–64, at 155.

77 Pollard, ‘“A Thing Like Death” ’, 104. The anaesthetist Anthony J. Carter has hypothesised that the wild neep (bryony) included in recipes for *dwale* (see below) had been substituted at some point for mandrake: Carter, ‘Dwale: An Anaesthetic from Old England’, *British Medical Journal*, 1999, *319*, 1623–1626, 1624.

78 WL MS 4338 (Johanna Saint John), fo. 56v; WL MS 8086 (Receipt Book), 113; Medical and Cookery Receipts, 1672-1680, MS Egerton 2214, BL, 59.

79 WL 373 (Jane Jackson), fo. 99r.

80 The Middle English word *dwale* means ‘a dazed or unconscious state’. These types of sleeping draughts are sometimes referred to by historians as anaesthetics because the aim was to render the patient unconscious of pain. However, the state induced may not have met the standard of the modern definition of anaesthesia, in which there should be a complete absence of pain. In the terminology of the time *dwale* was classified as a *stupefactive* because it dulled the patient’s senses. See Linda E. Voigts and Robert P. Hudson, ‘A Drynke þ at Men Callen Dwale to Make a Man to Slepe Whyle Men Keerven Hem: A Surgical Anesthetic from Late Medieval England’, in Sheila Campbell and David Klausner, eds, *Health, Disease and Healing in Medieval Culture* (London: Palgrave Macmillan, 1992), 34–56, at 36 and nt. 7.

81 Thomas Bonham, *The Chyrugians Closet: or, An Anitdotarie Chyrurgicall Furnished with Varietie and Choyce* (London, 1630), 244–45; Lupton, *A Thousand Notable Things*, 79. For an example of a similar sleeping draught in a fifteenth-century receipt book see Warren R. Dawson, ed, *A Leechbook or Collection of Medical Recipes of the Fifteenth Century*, 2nd edn (Kessinger, 2004), 262. Gwen Seabourne has found evidence of the use (or misuse) of this drug, or something similar, in England as early as the late thirteenth century: Gwen Seabourne, ‘Drugs, Deceit and Damage in Thirteenth-century Herefordshire: New Perspectives on Medieval Surgery, Sex and the Law’, *Social History of Medicine*, 2016, *30*, 255–76, at 258–62.

82 Voigts and Hudson, ‘A Drynke þ at Men Callen Dwale’, 35, 40–41, 43–44.

83 Andrew Wear, *Knowledge and Practice in Early Modern English Medicine, 1550-1680* (Cambridge: Cambridge University Press, 2000), ch. 8.

84 Georgina D. Hedesan, ‘Alchemy, Potency, Imagination: Paracelsus’s Theories of Poison’, in Grell and Cunningham, eds, *It All Depends*, 81–102, at 91–94; see also W. B. Deichmann, D. Henschler, B. Holmstedt and G. Keil, ‘What is there that is not Poison? A Study of the Third Defense by Paracelsus’, *Archives of Toxicology*, 1986, *58*, 207–13.

85 Oswald Croll, *Basilica Chymica, & Praxis Chymiatricae, or Royal and Practical Chemistry in Three Treatises*, trans. anon (London, 1670), 86. Similar sentiments can be found in Giambattista della Porta, *Natural Magick … Wherein Are Set Forth All the Riches and Delights of the Natural Sciences*, trans. anon (London, 1658), 217; and Angelus Sala, *Opiologia: or, A Treatise Concerning the Nature, Properties, True Preparation and Safe Use and Administration of Opium*, trans. Thomas Bretnor (London, 1618), 29. The *arcanum* was a marvellous medicine or elixir that cured all diseases.

86 See, for instance, the recipe for ‘Laudanum Anodinum’, which contains wine, henbane gum, unicorn horn, amber and potable gold, in Paracelsus, *A Hundred and Fouretene Experiments and Cures*, trans. John Hester (London, 1596), 20–21. While the invention of *laudanum* is attributed to Paracelsus, his version was unlikely to have borne much resemblance to later versions, and probably did not contain opium. His followers often used his name to promote their own remedies: Henry E. Sigerist, ‘Laudanum in the Works of Paracelsus’, *Bulletin of the History of Medicine*, 1941, *9*, 530–44.

87 Sala, *Opiologia*, sig. A4(r) – A5(v), B(v).

88 On *spagyria* and its importance in medicine see Lawrence M. Principe, *The Aspiring Adept: Robert Boyle and His Alchemical Quest* (Princeton, NJ: Princeton University Press, 1998), 9, 186–88.

89 The traditional argument for classifying opium as cold in the fourth degree was its observable effects upon a patient suffering from sleeplessness, and the danger of lethargy and death. Arguments for classifying it as a hot substance were, that it was bitter tasting and corrosive to the skin, it had a pungent smell and was darkish yellow in colour, and that it was observed to produce a sweat. See Sala, *Opiologia*, 17–26; Walter Harris, *Pharmcologia Anti-empirica, or, A Rational Discourse of Remedies Both Chymical and Galenical* (London, 1683), 194-196. On the methods of determining the classification of drugs see Michael McVaugh, ‘Determining a Drug’s Properties: Medieval Experimental Protocols’, *Bulletin of the History of Medicine*, 2017, *91* (Special Issue: Elaine Leong and Alisha Rankin, eds, *Testing Drugs and Trying Cures*), 183–209.

90 Sala, *Opiologia*, 14, 34–39.

91 See nts. 23, 24, 25, 26.

92 Thomas Willis, *Pharmaceutice Rationalis: or, An Exercitation of the Operations of Medicines in Humane Bodies*, 2 vols (1679), I. 150. Lawrence Principe, in his research into alchemy and its relationship to early chemistry, argues that Robert Boyle saw a distinction between the art of alchemy, which was conducted on the basis of knowledge of the principles of nature, and the ‘unphilosophical’ pharmacist, whose products could not be trusted because they were not based on sound understanding of the principles behind chymical processes: Principe, *The Aspiring Adept*, 30–35.

93 Bradley, *British Housewife*, 384–85.

94 Gideon Harvey, *A Treatise of Small-pox and Measles* (London, 1696), 154.

95 Bonet, *Guide to the Practical Physician*, 752.

96 Rankin, *Panaceia’s Daughters*, 142–47.

97 Raymond A. Anselment, ed, *The Remembrances of Elizabeth Freke, 1671-1714* (Cambridge: Cambridge University Press, 2001), 329–30.

98 WL MS 3500 (Mrs Meade), fo. 31r.

99 Cookery-Book, 17th/18th Century, MS 1795, WL, fo. 221v.

100 WL MS 1340 (Boyle Family), fo. 121r.

101 Nils-Otto Ahnfelt and Hjalmar Fors, ‘Making Early Modern Medicine: Reproducing Swedish Bitters’, *Ambix*, 2016, *63*, 162–83, at 172–75.

102 Robert Boyle, ‘An Account of the Two Sorts of the Helmontian Laudanum’, *Philosophical Transactions of the Royal Society*, 1674, *9*, 147–49; see correspondence with Dr Richard Lower and Daniel Coxe, in Michael Hunter, Antonio Clericuzio, and Lawrence M. Principe, eds, *The Correspondence of Robert Boyle*, 6 vols (London: Pickering and Chatto, 2001), II. 277–82, III. 247–51. Boyle believed that health was the highest endeavour of chymistry, see reference at nt. 88.

103 Sala, *Opiologia*, sig. [A5(v) – B(r)].

104 BL Lansdowne MS 57: No. 39 (Talbot letter).

105 Anselment, ed, *Remembrances of Elizabeth Freke*, 329–30.

106 Lady Sarah Cowper, Diary, vol. 2, 1703-1705, D/EP F30, Hertfordshire Archives and Local Studies, *Perdita Manuscripts*, Adam Matthews [amdigital.co.uk/primary-sources/Perdita-manusripts-1500-1700, accessed January 25, 2018], fo. 156r.

## Notes

Elizabeth Hunter is a Wellcome funded scholar at the Centre for the History of Emotions, Queen Mary, University of London. She has published articles in *The Seventeenth Century* (2021), *Reformation and Renaissance Review* (2013), and *Études Épistémè* (2015), and has contributed to collections on the history of medicine and emotion. Her most recent chapter appeared in James Kennaway and Rina Knoeff, eds, *Lifestyle and Medicine in the Enlightenment* (2020). She completed her DPhil at the University of Oxford.

